# Differential DNA methylation by early versus late parenteral nutrition in the PICU: a biological basis for its impact on emotional and behavioral problems documented 4 years later

**DOI:** 10.1186/s13148-021-01124-3

**Published:** 2021-07-27

**Authors:** An Jacobs, Fabian Güiza, Ines Verlinden, Karolijn Dulfer, Gonzalo Garcia Guerra, Koen Joosten, Sascha C. Verbruggen, Ilse Vanhorebeek, Greet Van den Berghe

**Affiliations:** 1 Clinical Division and Laboratory of Intensive Care Medicine, Department of Cellular and Molecular Medicine, KU Leuven, Herestraat 49, 3000 Leuven, Belgium; 2 Intensive Care Unit, Department of Pediatrics and Pediatric Surgery, Erasmus Medical Center, Sophia Children’s Hospital, Rotterdam, The Netherlands; 3 Department of Pediatrics, Intensive Care Unit, University of Alberta, Stollery Children’s Hospital, Edmonton, Canada

**Keywords:** PICU, Pediatric critical illness, Parenteral nutrition, DNA methylation, Long-term outcome, Neurocognitive development

## Abstract

**Background:**

The PEPaNIC multicenter randomized controlled trial (RCT) has shown that early administration of supplemental parenteral nutrition (early-PN) as compared with withholding PN for 1 week (late-PN) induced long-term internalizing, externalizing and total emotional/behavioral problems in critically ill children, as observed 4 years later. Early-PN was further shown to alter the methylation status of 37 CpG-sites in leukocyte DNA between admission and discharge from the pediatric intensive care unit (PICU). In a preplanned subanalysis of the PEPaNIC trial, we now investigated whether the altered methylation of these CpG-sites could statistically explain the negative impact of early-PN on emotion/behavior documented 4 years after PICU admission.

**Results:**

The combination of DNA methylation data and data on behavior 4 years after PICU admission was available for 403 of the 1440 patients (aged 0–17 years at PICU admission) who were included in the PEPaNIC RCT (192 early-PN and 211 late-PN patients). Mediation analyses with use of bootstrapped multivariable non-linear regression analyses adjusted for baseline risk factors revealed that the adverse alterations by early-PN in methylation of the 37 CpG-sites together statistically explained its harmful impact on internalizing, externalizing and total emotional/behavioral problems. When adding the methylation status of the 37 CpG-sites to the models, the explanatory power improved with a 1.710 to 1.851-fold increase, and the impact of the altered methylation status of the CpG-sites explained the impact of the randomization to early-PN versus late-PN.

**Conclusions:**

Abnormal DNA methylation induced by the early use of PN in the PICU provides a biological basis for its long-term harmful effect on emotion/behavior of critically ill children 4 years after PICU admission.

*Trial Registration* ClinicalTrials.gov NCT01536275, registered February 17, 2012, https://clinicaltrials.gov/ct2/show/NCT01536275.

**Supplementary Information:**

The online version contains supplementary material available at 10.1186/s13148-021-01124-3.

## Background

Nutritional support is an important aspect of the clinical management of critically ill children admitted to the pediatric intensive care unit (PICU). The enteral route for providing macronutrients is preferred [1], but feeding through mouth or via nasogastric tube is often not tolerated or impossible [2]. Therefore, supplemental parenteral nutrition (PN) is often initiated whenever enteral nutrition fails to reach the caloric targets. The multicenter ‘Pediatric Early versus Late Parenteral Nutrition in Critical Illness’ (PEPaNIC) randomized controlled trial (RCT) demonstrated that initiating supplemental PN within the first 24 h after admission (early-PN) was clinically inferior to postponing the initiation of supplemental PN until day 8 of PICU stay (late-PN), as patients in the early-PN group recovered more slowly and acquired more infections during PICU stay [3]. This nutritional strategy also appeared to contribute to long-term harm, as 2 years after PICU admission, early-PN patients showed worse parent/caregiver reported executive functioning (inhibitory control, working memory, meta-cognition and total executive functioning), externalizing behavioral problems and visual-motor integration [4]. Interestingly, a secondary analysis of the PEPaNIC RCT provided a possible molecular basis for the adverse effects of critical illness on long-term development and the role of nutritional management herein [5]. This large epigenome-wide study has shown that the administration of early-PN during critical illness altered the DNA methylation status of 37 CpG-sites. Several of the affected CpG-sites were located in genes involved in neuronal development, migration and plasticity, information processing, cerebral cortex development, attention deficit hyperactivity disorder, intellectual disability and neurodevelopment and neuron plasticity. Moreover, the early-PN-induced altered DNA methylation was found to statistically explain at least partially the early-PN-induced impaired neurocognitive outcomes documented 2 years later. As the long-term developmental effect of the nutritional strategy could change over time, and as clinical neurocognitive testing is more complete and more reliable from the age of 4 years onward, and since almost half of the patients were younger than 1 year at randomization, a 4-year follow-up study was also performed. This study has demonstrated a harmful effect of early-PN for development of internalizing, externalizing and total behavioral/emotional problems [6]. The aim of the current study was to investigate whether altered methylation of the 37 CpG-sites with the use of early-PN, as was previously identified, is also statistically explanatory for its negative impact on behavior documented 4 years after PICU admission.

## Results

**Fig. 1:**
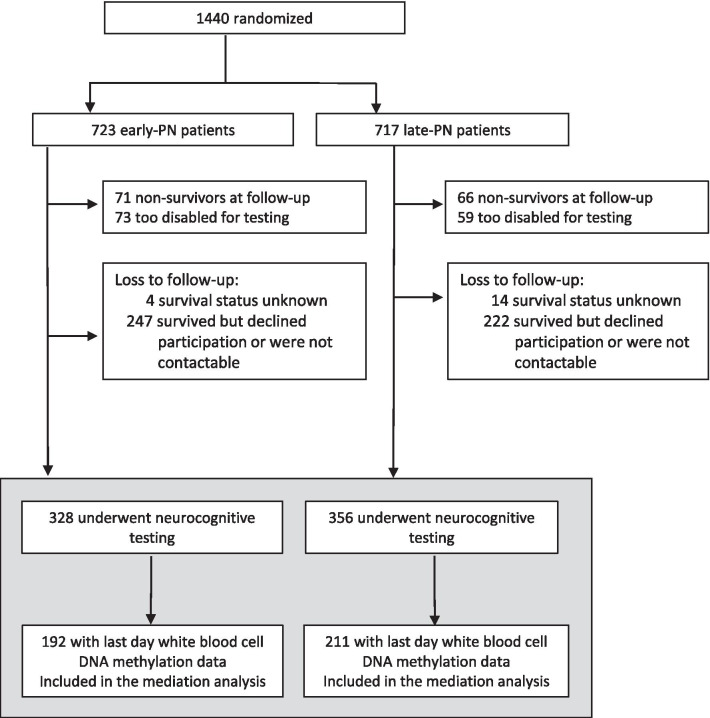
Consort diagram of study participants

Of the 1440 children who participated in the PEPaNIC study, 684 had been tested at 4-year follow-up. DNA methylation data were available for 403 of the 684 tested patients (192 early-PN and 211 late-PN patients, Fig. 1). Demographics and other patient characteristics upon PICU admission of both groups were comparable and are presented in Tables 1 and 2.

**Table 1:** Demographics of patients with DNA methylation analyzed on the last day of PICU stay

Demographics	Tested post-PICU population		
	Early-PN *N* = 192	Late-PN *N* = 211	*P* value^d^
Age at 4-year follow-up—year	7.6 (4.2)	7.5 (4.1)	0.74
Sex			
Male	111 (57.8%)	125 (59.2%)	0.77
Female	81 (42.2%)	86 (40.8%)	
Known non-Caucasian race^a^	13 (6.8%)	7 (3.3%)	0.10
Known non-European origin^a^	41 (21.4%)	30 (14.2%)	0.06
Known not exclusive Dutch or English language	49 (25.5%)	49 (23.2%)	0.59
Socioeconomic status			
Educational level parents/caregivers^b^			0.60
Educational level 1	6 (3.1%)	11 (5.2%)	
Educational level 1.5	17 (8.9%)	12 (5.7%)	
Educational level 2	43 (22.4%)	53 (25.1%)	
Educational level 2.5	27 (14.1%)	34 (16.1%)	
Educational level 3	55 (28.7%)	60 (28.4%)	
Educational level unknown	44 (22.9%)	41 (19.4%)	
Occupational level parents/caregivers^c^			0.10
Occupational level 1	0 (0.0%)	5 (2.4%)	
Occupational level 1.5	18 (9.4%)	27 (12.8%)	
Occupational level 2	33 (17.2%)	35 (16.6%)	
Occupational level 2.5	19 (9.9%)	16 (7.6%)	
Occupational level 3	34 (17.7%)	45 (21.3%)	
Occupational level 3.5	14 (7.3%)	14 (6.6%)	
Occupational level 4	31 (16.2%)	37 (17.5%)	
Occupational level unknown	43 (22.4%)	32 (15.2%)	

Data are n (%) or mean (SD)

*PICU* pediatric intensive care unit, *PN* parenteral nutrition, *SD* standard deviation

^a^Participants were classified according to race and geographical origin by the investigators. These classifications were performed to capture ethnical and regional differences in the frequency of consanguinity, which may adversely affect cognitive performance [7]

^b^The education level is described in Additional file 2

^c^The occupation level is described in Additional file 2

^d^*P* values were calculated with Wilcoxon Rank Sum tests for continuous data, and Chi square tests for proportions

**Table 2:** Baseline characteristics and acute outcomes of patients with DNA methylation analyzed on the last PICU-day

Characteristic	Tested post-PICU population		
	Early-PN *N* = 192	Late-PN *N* = 211	*P* value^f^
Infant (age < 1 year) at randomization	71 (37.0%)	91 (43.1%)	0.20
STRONGkids risk level ^a^			0.19
Medium	184 (95.8%)	196 (92.9%)	
High	8 (4.2%)	15 (7.1%)	
PeLOD score, first 24 h in PICU ^b^	23.7 (10.3)	24.1 (10.4)	0.62
PIM3 score ^c^	− 3.6 (1.3)	− 3.7 (1.1)	0.65
PIM3 probability of death—%^d^	6.1 (10.9)	4.5 (8.0)	0.65
Diagnostic category			0.44
Surgical			
Abdominal	7 (3.7%)	6 (2.8%)	
Burns	1 (0.5%)	0 (0.0%)	
Cardiac	109 (56.8%)	123 (58.3%)	
Neurosurgery-traumatic brain injury	17 (8.9%)	19 (9.0%)	
Thoracic	9 (4.7%)	11 (5.2%)	
Transplantation	0 (0.0%)	4 (1.9%)	
Orthopedic surgery-trauma	10 (5.2%)	5 (2.4%)	
Other	5 (2.6%)	8 (3.8%)	
Medical			
Cardiac	5 (3.7%)	5 (2.4%)	
Gastrointestinal–hepatic	1 (0.5%)	1 (0.5%)	
Oncologic–hematologic	0 (0.0%)	2 (1.0%)	
Neurologic	13 (6.8%)	10 (4.7%)	
Respiratory	8 (4.2%)	8 (3.8%)	
Other	7 (3.7%)	9 (4.3%)	
Malignancy	14 (7.3%)	12 (5.7%)	0.51
Diabetes	0 (0.0%)	0 (0.0%)	> 0.99
Syndrome^e^	15 (7.8%)	23 (10.9%)	0.28
Known parental smoking between birth and PICU admission	31 (16.1%)	42 (19.9%)	0.41
Duration of stay in the PICU—days	5.6 (11.7)	3.6 (3.5)	0.37
Patients who acquired a new infection in PICU	23 (12.0%)	9 (4.3%)	0.003
Duration of mechanical ventilatory support—days	3.1 (5.7)	2.2 (2.6)	0.66
Number of days with hypoglycemia < 40 mg/dl—days	0.1 (0.6)	0.2 (0.5)	0.11

Data are n (%) or mean (SD)

*BMI* body mass index, *PeLOD* pediatric logistic organ dysfunction score, *PICU* pediatric intensive care unit, *PIM3* pediatric index of mortality 3 score, *PN* parenteral nutrition, *SD* standard deviation

^a^Scores on the Screening Tool for Risk on Nutritional Status and Growth (STRONGkids) range from 0 to 5, with a score of 0 indicating a low risk of malnutrition, a score of 1–3 indicating medium risk, and a score of 4–5 indicating high risk [8]

^b^Pediatric Logistic Organ Dysfunction (PeLOD) scores range from 0 to 71, with higher scores indicating more severe illness [9]

^c^Pediatric Index of Mortality 3 (PIM3) scores, with higher scores indicating a higher risk of mortality [10]

^d^Pediatric Index of Mortality 3 (PIM3) probability of death, ranging from 0 to 100%, with higher percentages indicating a higher probability of death in PICU

^e^A pre-randomization syndrome or illness a priori defined as affecting or possibly affecting neurocognitive development (Additional file 2)

^f^*P* values were calculated with Wilcoxon Rank Sum tests for continuous data, and Chi square tests for proportions

**Fig. 2:**
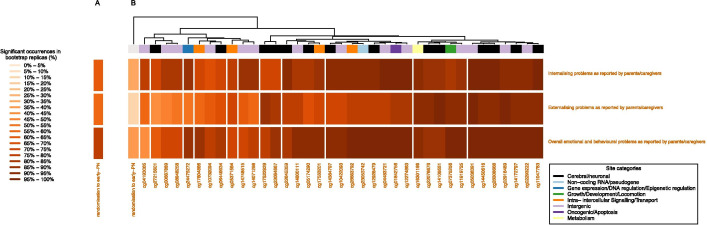
Visualization of the mediating role of the early-PN-induced alterations in DNA methylation in explaining impaired long-term emotional/behavioral outcomes with early-PN. This figure summarizes the results of the mediation analyses that were performed to investigate any role of early-PN-induced adversely altered leukocyte DNA methylation of 37 CpG-sites [5] in statistically explaining the harmful impact of early-PN on internalizing, externalizing and total behavioral problems identified at 4-year follow-up. All multivariable models resulted from random forest machine learning, with covariate significance levels obtained via permutation importance [11, 12], including the baseline risk factors age, center, race, sex, geographic origin, language, history of malignancy, a predefined “syndrome” (Additional file 2), diagnosis and severity of illness (PIM3 and PeLOD score), risk of malnutrition (STRONGkids score) and the randomized intervention, without (panel A) or with (panel B) the 37 differentially methylated CpG-sites evoked by early-PN [5]. The robustness of these results was evaluated in 100 bootstrapped replicates [13, 14]. Each row corresponds to the 100 bootstrap replicates of the multivariable non-linear models for the internalizing, externalizing and total behavioral problems. Columns correspond to the 37 CpG-sites that were differentially methylated by early-PN. Color intensity of the boxes reflects the frequency with which a CpG-site was found to be independently and significantly (*P* < 0.05) associated with the behavioral outcomes in the 100 bootstrapped replicated analyses, with darker orange colors corresponding to a higher frequency. Outcomes and CpG-sites were clustered based on these frequencies, with the clustering hierarchy shown in the dendrograms. The base of the column dendrogram is color-coded according to CpG-site functional classes “Cerebral/neuronal”, “Growth/Development/Locomotion”, “Metabolism”, “Gene expression/DNA regulation/Epigenetic regulation”, “Intra/intercellular Signaling/Transport”, “Non-coding RNA/pseudogene”, “Oncogenic/Apoptosis”, or “Intergenic”, as previously described [5]

In a non-linear model adjusted for baseline risk factors (age, center, sex, race, geographic origin, language, history of malignancy, a predefined syndrome, admission diagnosis, severity of illness upon PICU-admission, and risk of malnutrition), randomization to early-PN versus late-PN was found to be a significant predictor of the internalizing, externalizing, and total emotional/behavioral problems (respectively 63%, 59% and 80% of 100 replicated bootstrapped analyses) at 4-year follow-up of the tested patient cohort (Fig. 2A). Mediation analysis revealed that the early-PN-induced adversely altered DNA methylation of 37 CpG-sites statistically explained its harmful impact on the three behavioral outcomes (Fig. 2B). When adding the 37 CpG-sites to the multivariable models for the 3 behavioral outcomes, the explanatory power (optimism-corrected *R*^2^) improved with a 1.710 to 1.851-fold increase, and the impact of the altered methylation status of the CpG-sites replaced and outweighed the impact of the randomization to early-PN versus late-PN. Table 3 shows the details on the added explanatory power for the 3 long-term outcomes provided by the 37 differentially methylated CpG-sites. Unsupervised clustering based on the frequencies with which a CpG-site was found to be independently and significantly associated with the behavioral outcomes in the 100 bootstrapped replicated analyses revealed distinct patterns of association of CpG-site methylation status with internalizing versus externalizing behavior (Fig. 2B). The CpG-sites that were most often retained in the bootstrapped non-linear models as independently associated with the three outcomes, were cg14172797 in PRKCA (involved in memory, mood regulation and behavior), cg11047783 in KAT6B (involved in cerebral cortex development, attention deficit hyperactivity disorder (ADHD) and intellectual disability), cg14109551 in CEP85L (involved in brain tumors, ADHD and bipolar disorder), cg14450616 in PLD3 (involved in neuronal development, survival and neurotransmission, visual learning, memory and flexibility), cg26308668 in srGAP1 (involved in neuronal development and migration, linked to mental retardation), cg14364797 in FNBP1 (involved in neuronal network formation and information processing), cg22645359 in TCF7L2 (involved in Wnt signaling and related to neurodevelopment and plasticity of mature neurons), cg12928479 in NLRC5 (involved in neuroimmune and neuroinflammatory processes), cg22076676 in THADA (involved in apoptosis, neuroinflammation and multiple sclerosis), cg01842756 in RNF217 (involved in apoptosis), cg16301196 in PLA2G15 (involved in lipid metabolism), cg07375256 in ZSCAN25 (transcriptional regulation (DNA binding and protein–protein interactions); genetic variation in ZSCAN25 has been associated with body weight, hip and brachial circumference), cg26683792 in SLC35E1 (unknown, putative transporter), and several intergenic and non-coding CpG-sites. Similar results were obtained when the randomized intervention was excluded from the models.

**Table 3:** Added explanatory power by early-PN-induced altered DNA methylation in predicting more emotional/behavioral problems with early-PN

Outcome assessed at 4-years follow-up	Non-linear regression models			
	Without randomized intervention		With randomized intervention	
	*R*^2a^	Fold-increase in *R*^2b^	*R*^2c^	Fold-increase in *R*^2d^
Internalizing problems as reported by parents/caregivers	0.618	2.026	0.619	1.851
Externalizing problems as reported by parents/caregivers	0.603	1.813	0.603	1.710
Total problems as reported by parents/caregivers	0.613	1.868	0.614	1.771

All models were evaluated in 100 bootstrapped replicates. Baseline risk factors included age, center, race [7], gender, geographic origin [7], language, history of malignancy, diabetes, a predefined “syndrome” (Additional file 2), diagnosis and severity of illness (PIM3 and PeLOD) [9, 10], and risk of malnutrition (STRONGkids) [8]. *R*^2^ for non-linear models computed according to Rubin’s rules

*PN* parenteral nutrition

^a^Optimism-corrected *R*^2^ of multivariable non-linear random forest regression models for outcomes assessed at 4-years follow-up including 37 differentially methylated CpG-sites and baseline risk factors

^b^Optimism-corrected *R*^2^ fold-increase with respect to multivariable non-linear regression models including baseline risk factors only

^c^Optimism-corrected *R*^2^ of multivariable non-linear random forest regression models for outcomes assessed at 4-years follow-up including 37 differentially methylated CpG-sites, baseline risk factors and the randomized intervention

^d^Optimism-corrected *R*^2^ fold-increase with respect to multivariable non-linear random forest regression models only including baseline risk factors and the randomized intervention

## Discussion

We here showed that the methylation status of 37 CpG-sites within leukocyte DNA, which was previously shown to be altered by the use of early-PN as compared with late-PN in the PICU, at least in part statistically explained the harmful impact of early-PN on emotional/behavioral problems 4 years after PICU admission. The CpG-sites of which methylation status contributed the most to this effect on the emotional/behavioral problems were related to genes involved in pathways that are relevant for the development of these problems.

Several studies have shown that underfeeding and/or overfeeding in childhood can affect long-term health and development through induction of DNA methylation changes [15–17]. The early-PN-induced aberrant alterations in DNA methylation that we have identified in critically ill children [5] statistically explained at least part of its harmful effect with regard to emotional/behavioral problems 4 years after PICU admission. Among the strongest associations were several CpG-sites related to genes involved in mood regulation and behavior, behavioral disorders, but also in several cognitive functions, in nervous system development and function, among others. Interestingly, many of the CpG-sites that played the strongest mediating role for the harmful effect of early-PN on behavior at 4-year follow-up were the same as the ones that also played the strongest mediating role for the harmful effect of early-PN on the impaired executive functioning at 2-year follow-up [5]. This important finding provided further support for the clinical data that already suggested that normal executive functioning is essential to develop normal behavior later on in life [18, 19].

The identification of a biological basis for long-term developmental problems in critically ill children opens perspectives for prevention and treatment. Ideally, the epigenetic changes that arise during PICU stay should be prevented. This was found to be the case for a part of the DNA methylation changes which were induced by the use of early-PN and which could be avoided by omitting PN until beyond the first week of critical illness. However, the early-PN induced alterations in DNA methylation only accounted for 37 of the 159 previously identified CpG-sites that are altered by critical illness and/or its management [5]. Hence, other contributing factors must play a role and perhaps some of these are also modifiable.

A limitation of this study, and of the previous study that identified the de novo DNA methylation changes [5], relates to the use of leukocyte DNA in relation to potential tissue-specificity of DNA methylation changes. However, overlapping DNA methylation changes have been described for brain and leukocytes [20, 21] and we previously showed that 8 of the 10 CpG-sites differentially methylated by early PN versus late PN for which cross-tissue comparison was possible showed a significant correlation between blood and brain [5] (the threshold of significance not being reached for cg14109551 in CEP85L and cg22076676 in THADA, Additional file 1). Other limitations are the potential underestimation of the number of affected CpG-sites due to exclusion of all CpG-sites that were already differentially methylated upon PICU admission versus healthy children, the potential underestimation of the number of hypomethylated CpG-sites as the bisulfite conversion procedure does not discriminate between 5-methylcytosine or 5-hydroxymethylcytosine (which is a first step towards demethylation), and the reduced sample size of patients who were assessed for altered DNA methylation and were tested for neurocognitive development as compared with the original study cohort of patients. An additional limitation is the absence of assessment of effects of DNA methylation on corresponding gene expression, which also implies that confirmation with the two-step Mendelian randomization approach [22, 23] of an epigenetic mediating effect as suggested in this study was not possible. Also, we cannot exclude any role of post-randomization exposure, which remains to be investigated in future studies. Finally, we did not adjust for gestational age, which has been associated with differential methylation at birth, even at full term [24].

## Conclusions

We showed that aberrant DNA methylation evoked by the use of early-PN in the PICU, at least in part, mediated the harmful effect of early-PN on emotional/behavioral problems observed 4 years later. This finding provides new insight in a biological basis explaining the harm of early-PN in the long-term.

## Methods

### Patients and outcomes at 4-year follow-up

Between 2012 and 2015, 1440 critically ill children (from term newborns to children 17 years of age), were included in the multicenter PEPaNIC RCT performed at the University Hospitals Leuven, Belgium; the Erasmus-MC Sophia Children’s Hospital, Rotterdam, The Netherlands; and the Stollery Children’s Hospital, Edmonton, Alberta, Canada (ClinicalTrials.gov NCT01536275) [3]. After informed consent, children were randomly allocated to receiving supplemental parenteral nutrition (PN) within the first 24 h of PICU admission to reach caloric targets when enteral nutrition was insufficient (early-PN) or to postponing this initiation of supplemental PN until day 8 of PICU admission (late-PN). For most patients randomized to the late-PN group, this meant receiving no PN at all in view of discharge before day 8. Supplemental PN was discontinued when enteral nutrition reached 80% or more of the caloric target [3]. Parents gave informed consent to contact them for a 4-year follow-up study. Four years after inclusion, between 2016 and 2019, survival status was assessed and survivors were screened for eligibility to be tested neurocognitively [6]. If eligible, patients were contacted by phone to schedule a follow-up visit for medical and neurocognitive assessment, at the hospital or at the patients’ home. Parents or legal guardians, or patients when 18 years or older, gave written informed consent according to local regulations. The institutional review boards at each participating site approved this follow-up study (ML8052; NL49708.078; Pro00038098). As described in the clinical 4-year follow-up study [6], the follow-up visit consisted of measuring head circumference, body weight and height, and a clinical neurological examination to assess gross neurological abnormalities. Diagnosis of a somatic or psychiatric illness and hospital admissions were assessed via a structured interview with the parents or caregivers. Next, neurocognitive development was tested extensively by clinical age-appropriate tests, including intelligence quotient, visuomotor integration, memory, alertness and motor coordination. Finally, parents or caregivers were asked to complete questionnaires on executive functioning and emotional and behavioral problems of the child. A total of 684 patients, 328 early-PN and 356 late-PN, and for comparison 369 matched healthy control children were assessed [6]. As inability to fully complete the neurocognitive test battery may indicate poor neurocognitive function and thus introduce bias [4], missing values were imputed by chained equations, with use of all available data per individual [6, 25, 26]. Bias and instability of the imputation model was minimized by only including outcomes with ≤ 30% missing data [25, 26]. The number of imputation models was set at 31 to avoid loss of statistical power [25, 26]. Multivariable linear regression analyses performed on the 31 imputed datasets revealed significantly more internalizing, externalizing, and total emotional/behavioral problems in the early-PN group as compared with the late-PN group, adjusted for baseline risk factors including age, center, sex, race, geographic origin, language, hand preference, history of malignancy, a predefined syndrome (Additional file 2), educational and occupational status of the parents/caregivers (Additional file 2), admission diagnosis, severity of illness upon PICU-admission (PIM3 and PeLOD score), risk of malnutrition (STRONGkids score), and parental smoking behavior prior to PICU-admission. For this developmental domain, the difference between late-PN patients and healthy controls was no longer present. An extended description of clinical tests, questionnaires, statistical analyses and results of the PEPaNIC 4-year follow-up study has been published elsewhere [6]. Of particular relevance for the present study is the assessment of emotional and behavioral problems with use of the Child Behavior Checklist (CBCL 1.5–5 years or CBCL 6–18 years) [27, 28]. This checklist interrogates internalizing, externalizing and total emotional and behavioral problems [6]. Internalizing problems are evidenced by anxious and depressive symptoms, and by social withdrawal, which are consequences of over-controlling behavior. Externalizing problems are externally directed problems that affect the environment and present themselves in aggressive and delinquent behavior, resulting in conflicts with others. The total score for the emotional and behavioral problems includes apart from internalizing and externalizing behavioral problems, also sleep problems for younger children, and social, thinking, and attention problems for older children.

### Assessing the role of the early-PN-induced altered methylation of 37 CpG-sites in statistically explaining its negative impact on developmental outcome at 4-years follow-up

Via a genome-wide DNA methylation analysis with use of the Infinium® HumanMethylation EPIC BeadChip (Illumina Inc., San Diego, CA), we have previously identified 37 CpG-sites in leukocyte DNA of which abnormal de novo alterations in methylation status were evoked by early-PN as compared with late-PN [5]. Details on the identification of these 37 CpG-sites are provided in Additional file 2 and information on the location of the CpG-sites, gene-related protein functions, and direction of change is provided in Additional file 1. In analogy with the earlier 2-year follow-up study [5], a mediation analysis was performed to investigate any role of the differential methylation status of the 37 CpG-sites in statistically explaining the impact of the randomized intervention on the emotional and behavioral 4-year follow-up outcomes. To this end, we performed multivariable non-linear regression analyses using a random forest technique, adjusted for baseline risk factors [age, center, sex, race, geographic origin, language, history of malignancy, a predefined syndrome (Additional file 2), admission diagnosis, severity of illness upon PICU-admission (PIM3 and PeLOD score) and risk of malnutrition (STRONGkids score)], and further for the methylation status of those 37 CpG-sites. Random forests are a collection of hundreds of decision trees, each built on resampled data and introducing additional randomness at each tree node by allowing only a random subset of predictors to be evaluated in selecting the best split for that node [12]. We used this technique to be able to take into account non-linear interactions between the intervention and the CpG-sites or amongst the CpG-sites, as it is known that disruption of epigenetic networks is a key pathogenic mechanism of intellectual and developmental disability [29]. Models including the CpG-sites were built with and without addition of the randomized intervention. Covariate significance levels obtained via permutation importance were used [10]. The technique does not offer the possibility to obtain a unique regression coefficient for each covariate, but the variable importance and the derived permutation-based p-values allow to quantify the relative contribution of each predictor. The robustness of the results was evaluated by 100 bootstrapped replicates and the fold-change in optimism-corrected *R*^2^ between models with and without addition of the CpG-sites differentially methylated by early-PN as compared with late-PN as covariates was calculated [30]. The optimism-corrected *R*^2^ refers to the classical coefficient of determination but with an adjustment consisting of subtracting the expected optimism from using a bootstrapped approach, since the performance measures would otherwise be biased towards values obtained from overfitted models. The combination of optimism-corrected *R*^2^ and p-values for the mediators (CpG sites) and for the intervention (feeding strategy) allow showing substantial contribution of the mediating path in the total effect of the intervention on the outcome. Heatmaps with dendrograms were used to visualize and summarize the findings.

Data are presented as means and standard deviations (SD), or numbers and proportions and were compared among groups with use of Wilcoxon Rank Sum tests for continuous data, and Chi square tests for proportions. Statistical analyses were performed with use of R version 3.5.3, MICE versions 3.4.0 and 3.6.0, and JMP© version 14.0.0 (SAS Institute, Inc, Cary, NC). Two-sided P-values at or below 0.05 were considered statistically significant.

## Supplementary Information

**Additional file 1.** Information on the location of the 37 CpG-sites with early PN-induced altered methylation, gene-related protein functions, and direction of change.

**Additional file 2.** Definition of ‘syndrome’, educational and occupational level of parents and description of the identification of early-PN induced altered DNA methylation in 37 CpG-sites.

## Data Availability

Data sharing will be considered only on a collaborative basis with the principal investigators, after evaluation of the proposed study protocol and statistical analysis plan.

## Abbreviations

ADHD: Attention deficit hyperactivity disorder
PN: Parenteral nutrition
PEPaNIC: Pediatric early versus late parenteral nutrition in critical illness
RCT: Randomized controlled trial
DNA: Deoxyribonucleic acid
PICU: Pediatric intensive care unit
CBCL: Child behavior checklist
PIM3: Pediatric index of mortality 3 score
PeLOD: Pediatric logistic organ dysfunction score
STRONGkids: Screening tool for risk on nutritional status and growth
