# Supplementary material for: Differential DNA methylation by early versus late parenteral nutrition in the PICU: a biological basis for its impact on emotional and behavioral problems documented 4 years later
Source: Clin Epigenetics. 2021 Jul 27;13:146. doi: 10.1186/s13148-021-01124-3 (PMC8314560; doi:10.1186/s13148-021-01124-3)
Supplement: Supplementary file 1 — Additional file 1. Information on the location of the 37 CpG-sites with early PN-induced altered methylation, gene-related protein functions, and direction of change. [file 13148_2021_1124_MOESM1_ESM.docx]

**Additional Table 1. CpG-sites differentially methylated by early-PN versus late-PN**

| **CpG-site** | **Gene symbol** | **Gene-related protein function** | **Gene section** | **More methylated in** |
| --- | --- | --- | --- | --- |
|  |  |  |  |  |
| cg10732094 ^a^ |  |  | Intergenic | Control |
| cg26308668 | SRGAP1 | regulating neuronal development and migration; mental retardation; autism; schizophrenia | Intron | Control |
| cg06449934 ^a^ | GPER1 | numerous intracellular signaling pathways among the cardiovascular, endocrine, reproductive, immune and central nervous systems; regulation of hippocampal memory and cognition; social and spatial recognition learning; working memory; synaptic transmission; anxiety | 5' UTR | Control |
| cg08948258 |  |  | Intergenic | Control |
| cg10422093 |  |  | Intergenic | Control |
| cg02918489 |  |  | Intergenic | Control |
| cg17522929 | PKM | glycolysis; transcriptional activation; caspase-independent cell death of tumor cells; may mediate metabolic effects of thyroid hormone; involved in bacterial pathogenesis (adherence of bacteria to human cells); brain development; neuronal differentiation | 5' UTR/ Intron | Control |
| cg04483721 |  |  | Intergenic | Control |
| cg14364797 ^a^ | FNBP1 | regulation of the actin cytoskeleton; spine formation/neurite branching; neuronal network formation; information processing | 3' UTR | Patient |
| cg14109551 ^b^ | CEP85L | brain tumors; attention deficit hyperactivity disorder (ADHD); bipolar disorders | Intron | Control |
| cg05174290 | ATAD2B | chromatin-related function; neuronal differentiation; tumor progression | Intron/ Non-coding | Patient |
| cg26683792 | SLC35E1 | unknown (putative transporter) | Intron | Patient |
| cg14172797 | PRKCA | many different cellular processes, such as cell proliferation, differentiation and apoptosis, cell cycle checkpoint, and cell volume control; cancer development; episodic remembering (memory); mood regulation; behaviour; choroid gliomas; highly expressed in brain | Intron | Control |
| cg16301196 | PLA2G15 | hydrolyzing lysophosphatidylcholine to glycerophosphorylcholine and a free fatty acid; phospholipid degradation | 3' UTR | Control |
| cg14450616 | PLD3 | hydrolysis of membrane phospholipids and processing of amyloid-beta precursor protein; neuronal development and survival, neurotransmission; visual learning, memory, speed and flexibility; Alzheimer’s disease; highly expressed in the brain | 5' UTR | Control |
| cg17533201 | RAB11FIP4 | regulating endocytic traffic; cytokinesis | Intron | Control |
| cg14071298 |  |  | Intergenic | Patient |
| cg07375256 ^a^ | ZSCAN25 | transcriptional regulation (DNA binding and protein-protein interactions); genetic variation in ZSCAN25 has been associated with body weight, hip and brachial circumference. | Intron | Patient |
| cg01842756 | RNF217 | apoptosis signaling | Intron/ Non-coding | Control |
| cg22076676 ^b^ | THADA | apoptosis; adaptive thermogenesis; homeostasis; neuroinflammation and multiple sclerosis | Intron/ Non-coding | Control |
| cg11047783 | KAT6B | transcriptional regulation; cerebral cortex development; cognition; ADHD; intellectual disability; microcephaly and growth regulation | Intron | Control |
| cg12928479 | NLRC5 | regulating the NF-kappa-B and type I interferon signaling pathways; immunity; neuroimmune and neuroinflammatory processes | Exon | Control |
| cg05371584 | TBC1D8 | unknown (putative role in GTPase-activation of Rab family proteins) | Intron/ Non-coding | Control |
| cg00687889 |  |  | Intergenic | Control |
| cg23053742 ^a^ | LACTB2-AS1 | non-coding RNA | Non-coding | Patient |
| cg12274883 |  |  | Intergenic | Control |
| cg14748515 |  |  | Intergenic | Control |
| cg14800111 |  |  | Intergenic | Control |
| cg04193065 ^a^ |  |  | Intergenic | Control |
| cg24475272 ^a^ | SETD7 | histone methylation, with impact on transcriptional activation of genes such as collagenase or insulin | 3' UTR/ Intron | Control |
| cg17804886 ^a^ | RASA3 | negatively regulating the Ras signaling pathway | 5' UTR/ Intron | Patient |
| cg11919725 |  |  | Intergenic | Patient |
| cg22645359 | TCF7L2 | transcriptional regulation in the Wnt signaling pathway; blood glucose homeostasis; related to neurodevelopment and plasticity of mature neurons; memory, visual motor/fine motor function, emotional functioning, behaviour and speech; schizophrenia; ADHD | 3' UTR/ Intron | Control |
| cg05038391 |  |  | Intergenic | Control |
| cg27215601 | NSMCE2 | nuclear transport, transcription, chromosome segregation and DNA repair; key role in genome maintenance, suppression of mitotic recombination; dwarfism; bipolar disorder | Intron/ Non-coding | Patient |
| cg23084667 | GRAP2 | leukocyte-specific protein-tyrosine kinase signaling; RET signaling which is involved in brain development and maturation of dopaminergic neurons | 5' UTR | Patient |
| cg02293222 |  |  | Intergenic | Control |
|  |  |  |  |  |

Source of gene-related protein function: Entrez Gene Summary at Pubmed.com and UniProt database (www.uniprot.org), complemented with updated literature searches for links with brain and development.

^a,b^: Only for 10 of the 37 CpG sites a cross-tissue blood-brain comparison of methylation status was possible with use of the BECon tool or the Brain Epigenomics blood brain DNA methylation comparison tool, showing a significant correlation between blood and brain for 8 (^a^) but not for the other 2 (^b^) [1-4].

Abbreviations: ADHD, attention deficit hyperactivity disorder; ATAD2B, ATPase Family AAA Domain Containing 2B; CEP85L, Centrosomal Protein 85 Like; DNA, deoxyribonucleic acid; FNBP1, Formin Binding Protein 1; GPER1, G Protein-Coupled Oestrogen Receptor 1; GRAP2, Growth factor Receptor-bound Protein 2-Related Adaptor Protein 2; KAT6B, Lysine Acetyltransferase 6B; LACTB2-AS1, Lactamase Beta 2 Antisense ribonucleic acid 1; NLRC5, NLR Family CARD Domain Containing 5; NSMCE2, Non-SMC Element 2 Methyl Methanesulfonate Sensitivity Gene 21 Homolog; PKM, Pyruvate Kinase Muscle; PLA2G15, Phospholipase A2 Group XV; PLD3, Phospholipase D Family Member 3; PRKCA, Protein Kinase C Alpha; RAB11FIP4, RAB11 Family Interacting Protein 4; RASA3, RAS P21 Protein Activator 3; RNA, ribonucleic acid; RNF217, Ring Finger Protein 217; SETD7, SET Domain Containing Lysine Methyltransferase 7; SLC35E1, Solute Carrier Family 35 Member E1; SRGAP1, SLIT-ROBO Rho GTPase Activating Protein 1; TBC1D8, TBC1 Domain Family Member 8; TCF7L2, Transcription Factor 7 Like 2; THADA, Thyroid Adenoma-Associated Protein; UTR, untranslated region; ZSCAN25, Zinc Finger And SCAN Domain Containing 25.

1. Güiza F, Vanhorebeek I, Verstraete S, Verlinden I, Derese I, Ingels C, et al. Effect of early parenteral nutrition during paediatric critical illness on DNA methylation as a potential mediator of impaired neurocognitive development: a pre-planned secondary analysis of the PEPaNIC international randomised controlled trial. Lancet Respir Med 2020;8:288-303
2. Edgar RD, Jones MJ, Meaney MJ, Turecki G, Kobor MS. BECon: a tool for interpreting DNA methylation findings from blood in the context of brain. Transl Psychiatry 2017;7:e1187
3. https://redgar598.shinyapps.io/BECon/ [Accessed May 20th, 2019]
4. https://epigenetics.essex.ac.uk/bloodbrain/ [Accessed May 20th, 2019]
