# Supplementary material for: Differential DNA methylation by early versus late parenteral nutrition in the PICU: a biological basis for its impact on emotional and behavioral problems documented 4 years later
Source: Clin Epigenetics. 2021 Jul 27;13:146. doi: 10.1186/s13148-021-01124-3 (PMC8314560; doi:10.1186/s13148-021-01124-3)
Supplement: Supplementary file 2 — Additional file 2. Definition of ‘syndrome’, educational and occupational level of parents and description of the identification of early-PN induced altered DNA methylation in 37 CpG-sites. [file 13148_2021_1124_MOESM2_ESM.docx]

**Additional Methods**

**Definition of “Syndrome”**

A pre-randomisation syndrome or illness *a priori* defined as affecting or possibly affecting neurocognitive development, and which is subdivided in the following categories [1]:

- - Genetically confirmed syndrome or pathogenic chromosomal abnormality
  - Clearly defined syndrome, association or malformation without (identified) genetic aberration
  - Polymalformative syndrome of unknown etiology
  - Clear auditory or visual impairment without specified syndrome
  - Congenital hypothyroidism due to thyroid agenesis
  - Brain tumor or tumor with intracranial metastatic disease
  - Pediatric psychiatric disorder (e.g. autism spectrum disorder, (treatment for) attention deficit hyperactivity disorder)
  - Severe medical disorder, not primarily neurologic, but suspected to alter psychomotor and/or mental performance
  - Severe neonatal problem (e.g. severe asphyxia)
  - Severe craniocerebral trauma or near-drowning
  - Severe infectious encephalitis or drug-induced encephalopathy
  - Infectious meningitis, encephalitis or Guillain-Barré
  - Resuscitation and/or need for extracorporeal membrane oxygenation prior to randomization
  - Severe convulsions or stroke prior to randomization

**Definition of educational and occupational level of parents**

**Educational level of parents [1]**

The education level is the average of the paternal and maternal educational level, and calculated based upon the 3-point scale subdivisions as made by the Algemene Directie Statistiek (Belgium; statbel.fgov.be/nl/) and the Centraal Bureau voor de Statistiek (The Netherlands; statline.cbs.nl): Low (=1), middle (=2) and high (=3) educational level.

**Occupational level of parents [1]**

The occupation level is the average of the paternal and maternal occupation level, which is calculated based upon the International Isco System 4-point scale for professions [2]. In case one of the parents filled in two jobs in the questionnaire, the highest Isco code level was used. In case “unemployed”, “disabled”, “student”, or “housewife/houseman” was filled in, an Isco code level of 1 was given to that parent. When the parents described their profession as “employee”, “worker”, “liberal profession”, or “retired”, they were given an Isco code level of 2.

**Identification of early-PN induced altered DNA methylation in 37 CpG-sites**

*DNA extraction, bisulfite conversion, genome-wide DNA methylation analysis, and quality assessment*

During the PEPaNIC-RCT, blood cell samples were collected from patients upon PICU admission and at PICU discharge, and for comparison from matched healthy children, immediately after placement of an intravenous catheter prior to minor elective surgery [3]. After extraction and bisulfite-conversion of genomic DNA, a genome-wide DNA-methylation analysis was performed with use of the Infinium® HumanMethylation EPIC BeadChip (Illumina Inc., San Diego, CA). This microarray interrogates >850.000 CpG-sites and spans >99% of genes in the Reference Sequence (RefSeq) database [4]. Methylation β-values, ranging from 0 (no methylation) to 1 (full methylation), and M-values, which are the log2 ratios of the intensities of methylated probes versus unmethylated probes [5], were obtained after functional normalization of the raw intensities. Quality of the DNA methylation data was assessed with evaluation of the bi-peak curve of the M-value distribution among the samples in the low- and high-end range, and with principal component analysis (PCA) that assessed the variance in the dataset. Probes with a mean detection p-value >0.01 were excluded, to ensure that signals were expressed above the background defined by negative control probes, as were probes on X and Y chromosomes and probes spanning single nucleotide polymorphisms [6]. Of the extracted leukocyte DNA samples, 814 last PICU day samples, 694 PICU admission samples, and 351 samples from healthy children passed all quality controls [3].

*Identification of CpG-sites that became differentially methylated during PICU stay*

As described earlier [3], in order to investigate whether DNA methylation was altered between PICU admission and discharge, M-values were compared between patients upon PICU admission and healthy controls with ANOVA, applying a false discovery rate (FDR) <0·05 to account for multiple testing inherent to genome-wide analyses [7]. All identified CpG-sites that were differentially methylated between patients and healthy controls were discarded for further analyses, as these differences reflect pre-morbid conditions and illness-induced alterations in leukocyte composition [8-12]. Subsequently, the degree of DNA methylation in the remaining CpG-sites was compared with ANOVA and FDR<0.05, between patients on the last PICU day and healthy controls. This analysis identified 159 CpG-sites as methylated differently in patients on the last PICU day than in matched controls, which are thus ‘*de novo*’ DNA methylation changes that arise in patients during PICU stay [3].

*Identification of de novo differential methylation of CpG-sites by early-PN versus late-PN*

To investigate which of the identified 159 CpG-sites became differentially methylated by early-PN versus late-PN on the last PICU day in patients, multivariable linear regression analyses were performed adjusting for baseline risk factors [age, center, race, gender, geographic origin, language, history of malignancy, diabetes, predefined syndrome, diagnosis and severity of illness (PIM3 and PeLOD score), and risk of malnutrition (STRONGkids score)]. This analysis was repeated in 100 bootstrap samples and the result declared “robust” if the association (P<0.05) was present in at least 50 [13,14]. These analyses showed that early-PN, as compared with late-PN, contributed to the abnormal methylation status of 37 of the 159 CpG-sites [3].

**References**

1. Verstraete S, Verbruggen SC, Hordijk JA, Vanhorebeek I, Dulfer K, Güiza F, et al. Long-term developmental effects of withholding parenteral nutrition for 1 week in the paediatric intensive care unit: a 2-year follow-up of the PEPaNIC international, randomised, controlled trial. Lancet Respir Med 2019; 7:141-153
2. <http://www.ilo.org/public/english/bureau/stat/isco/>.
3. Güiza F, Vanhorebeek I, Verstraete S, Verlinden I, Derese I, Ingels C, et al. Effect of early parenteral nutrition during paediatric critical illness on DNA methylation as a potential mediator of impaired neurocognitive development: a pre-planned secondary analysis of the PEPaNIC international randomised controlled trial. Lancet Respir Med 2020; 8:288-303.
4. McCartney DL, Walker RM, Morris SW, McIntosh AM, Porteous DJ, Evans KL, et al. Identification of polymorphic and off-target probe binding sites on the Illumina Infinium MethylationEPIC BeadChip. Genom Data 2016; 9:22-24.
5. Du P, Zhang X, Huang CC, Jafari N, Kibbe WA, Hou L, et al. Comparison of Beta-value and M-value methods for quantifying methylation levels by microarray analysis. BMC Bioinformatics 2010; 11:587.
6. Chen YA, Lemire M, Choufani S, Butcher DT, Grafodatskaya D, Zanke BW, et al. Discovery of cross-reactive probes and polymorphic CpGs in the Illumina Infinium HumanMethylation450 microarray. Epigenetics 2013; 8:203-209.
7. Reiner A, Yekutieli D, Benjamini Y. Identifying differentially expressed genes using false discovery rate controlling procedures. Bioinformatics 2003; 19:368-375.
8. Adalsteinsson BT, Gudnason H, Aspelund T, Harris TB, Launer LJ, Eiriksdottir G, et al. Heterogeneity in white blood cells has potential to confound DNA methylation measurements. PLoS One 2012; 7:e46705.
9. Houseman EA, Molitor J, Marsit CJ. Reference-free cell mixture adjustments in analysis of DNA methylation data. Bioinformatics 2014; 30:1431-1439.
10. Jiang R, Jones MJ, Chen E, Neumann SM, Fraser HB, Miller GE, et al. Discordance of DNA methylation variance between two accessible human tissues. Sci Rep 2015; 5:8257.
11. Muszynski JA, Thakkar R, Hall MW. Inflammation and innate immune function in critical illness. Curr Opin Pediatr 2016; 28:267-273.
12. Rahmani E, Zaitlen N, Baran Y, Eng C, Hu D, Galanter J, et al. Sparse PCA corrects for cell type heterogeneity in epigenome-wide association studies. Nat Methods 2016; 13:443-445.
13. Austin PC, Tu JV. Bootstrap methods for developing predictive models. Am Stat 2004; 58:131-137.
14. Sauerbrei W, Schumacher M. A bootstrap resampling procedure for model-building - Application to the Cox regression-model. Statistics in Medicine 1992; 11:2093-2109.
